# In Search of Alternate Implantation Sites for Pacemakers and Defibrillators: Pacing Along Creativity and Innovation

**DOI:** 10.19102/icrm.2019.100406

**Published:** 2019-04-15

**Authors:** Arash Aryana

**Affiliations:** ^1^Mercy General Hospital and Dignity Health Heart and Vascular Institute, Sacramento, CA, USA

**Keywords:** Axillary fossa, cardiac pacing, cardiovascular implantable electronic device, pacemaker

In this month’s issue of *The Journal of Innovations in Cardiac Rhythm Management*, Kloosterman et al.^1^ report on the use of the axillary fossa as an alternate site for the implantation of cardiovascular implantable electronic devices (CIEDs). The authors report their experience using this approach in a cohort of five individuals consisting largely of elderly, emaciated patients with significant thinning of the musculocutaneous tissue, including two patients undergoing a pacemaker generator replacement, two patients receiving de novo pacemaker implants, and one patient undergoing a cardiac resynchronization therapy defibrillator generator replacement. Briefly, this interesting technique involves establishing venous access using the conventional approach. Subsequently, the lead(s) are tunneled and connected to the generator, which is implanted in a pocket created within the axillary fossa. Based on the authors’ experience, all five implants yielded favorable outcomes with the exception of a case of transient postoperative numbness in the ipsilateral arm with no pain or functional mobility impairment that eventually resolved without intervention.

The birth of CIED implantation and surgery are intertwined as, in 1957, C. Walton Lillehei, a cardiothoracic surgeon at the University of Minnesota, and coworkers first developed a myocardial wire for postoperative pacing.^2^ This marked the first implant of an electronic medical device in a human. Prior to that, cardiac pacing had been attempted clinically and performed in 1926 by Mark Cowley Lidwell, an Australian anesthesiologist, who used this approach to resuscitate a newborn baby at the Crown Street Women’s Hospital in Sydney, Australia.^3^ A few years later, Albert Hyman, a cardiologist from New York, together with his brother, Charles, constructed an electromechanical device that operated using a hand crank apparatus that generated and directed electrical impulses to a patient’s right atrium via a needle electrode inserted intercostally.^2^ This so-called “artificial pacemaker” was tested in several animals and at least one human.^4^ However, it never gained widespread acceptance by the medical community, which largely opposed Hyman in his attempts to popularize the use of this invention. It was not until 1958 when Rune Elmqvist and Åke Senning from the Karolinska Institute in Sweden implanted the first “implantable” pacemaker in Arne Larsson, a patient suffering from complete heart block and recurrent bouts of Stokes–Adams syncope.^2^ The procedure was performed by way of a left thoracotomy to implant the electrodes onto the myocardium, which were then tunneled to the pacemaker generator and inserted in the abdominal wall.^5^ The generator was molded after a popular shoe polish can (Kiwi^®^; S. C. Johnson & Son, Racine, WI, USA) and weighed close to 0.50 lbs. Within eight hours of the implant, Larsson required a generator replacement. Ultimately, he went on to undergo more than 20 pacemaker replacements and eventually outlived both his surgeon and pacemaker engineer.

These early and crude attempts were eventually followed by monumental technological advances that further paved the road for the development of not only modern-day pacemakers but also implantable cardioverter-defibrillator and cardiac resynchronization therapy devices. Over time, large external alternating current–powered generators with extension cords gave way to progressively smaller, contemporary battery-operated CIEDs. The pulse generators were initially implanted in the abdomen. However, with progressive reductions in size, they were redesigned for implantation within the infraclavicular pectoral space for simplicity and ease of subsequent access. Though this now represents the most common site for placement of a transvenous CIED, in some patients, it might not represent the most optimal location. The surgical scar at this site is often highly visible and, depending on the patient’s body habitus, the prominent contour of the generator may be rather conspicuous underneath the skin and the superficial tissue. Additionally, the cosmetic results in some cases can be less than satisfactory if the scar stretches over time, becoming thin and translucent, while, in other patients prone to this, it might result in keloid formation. Therefore, alternate CIED implantation sites such as the axillary fossa have been proposed and previously explored by several operators.^6–11^

Specifically, there appear to be several benefits to an axillary fossa CIED implant. First, the surgical scar and generator contour are mostly hidden from clear view, while the device is still maintained in an easily accessible pocket site for subsequent generator replacements.^11^ Second, the axilla is not in direct contact with external elements such as undergarments, other clothing items, or seat belts, which have the potential to cause discomfort and even wound stress. Third, lead mobility and torqueing stress are thought to be minimal within the axillary fossa. Another theoretical benefit may be that, in certain patients, an axillary fossa approach may improve the defibrillation vector. As de novo CIED implants and the need for multiple generator replacements become increasingly prevalent in the elderly population, alternate implantation sites such as the axillary fossa will likely become more pervasive and perhaps even necessary over time. Another interesting cohort to consider is the pediatric patient population, which often requires multiple generator replacements and lead revisions over a span of many years, which could lead to undesirable scar expansion and related changes. Additionally, several quality of life studies investigating individuals with CIED implants have identified concerns surrounding self-image as a major source of distress in these patients.^12,13^ Along these lines, Brown et al. evaluated the quality of life experiences reported by patients with visible scars and concluded that the vast majority were unhappy with their scars’ appearances due to perceived stigma.^14^ These concerns can be further augmented in the younger pediatric population given their growing and evolving self-image. Hence, it seems particularly reasonable to consider alternate implantation sites in pediatric patients receiving CIEDs. Still, long-term data on the risk and outcomes of infection, multiple device replacements, and lead extraction efficacy are currently lacking for the axillary fossa implant method, thus warranting further investigation. Moreover, there are also limited data on the safety of this approach with regard to vascular and nerve injury, especially given the close proximity of this space to the intercostobrachial nerve and the brachial plexus.

In summary, the history of CIED invention and implantation is an inspiring story of the initiative, ingenuity, and innovation displayed by the pioneers who advanced this field in the face of challenge and skepticism. As such, it represents a unique blend between creativity, technological advancement, and the art of medicine, which continues to evolve to this day, as illustrated well by the manuscript from Kloosterman et al. in this issue of *The Journal of Innovations in Cardiac Rhythm Management*.
